# EMG Measurement with Textile-Based Electrodes in Different Electrode Sizes and Clothing Pressures for Smart Clothing Design Optimization

**DOI:** 10.3390/polym12102406

**Published:** 2020-10-19

**Authors:** Siyeon Kim, Sojung Lee, Wonyoung Jeong

**Affiliations:** Human Convergence Technology R&D Department, Korea Institute of Industrial Technology, 143 Hanggaul-ro, Sangrok-gu, Ansan-si 15588, Gyeonggi-do, Korea; siyeonkim@kitech.re.kr (S.K.); jungyee814@kitech.re.kr (S.L.)

**Keywords:** textile based electrodes, electromyography, EMG, clothing pressure, electrode size, clothing design optimization

## Abstract

The surface electromyography (SEMG) is one of the most popular bio-signals that can be applied in health monitoring systems, fitness training, and rehabilitation devices. Commercial clothing embedded with textile electrodes has already been released onto the market, but there is insufficient information on the performance of textile SEMG electrodes because the required configuration may differ according to the electrode material. The current study analyzed the influence of electrode size and pattern reduction rate (PRR), and hence the clothing pressure (*P_c_*) based on in vivo SEMG signal acquisition. Bipolar SEMG electrodes were made in different electrode diameters Ø 5–30 mm, and the clothing pressure ranged from 6.1 to 12.6 mmHg. The results supported the larger electrodes, and *P_c_* showed better SEMG signal quality by showing lower baseline noise and a gradual increase in the signal to noise ratio (SNR). In particular, electrodes, Ø ≥ 20 mm, and *P_c_* ≥ 10 mmHg showed comparable performance to Ag-Ag/Cl electrodes in current textile-based electrodes. The current study emphasizes and discusses design factors that are particularly required in the designing and manufacturing process of smart clothing with SEMG electrodes, especially as an aspect of clothing design.

## 1. Introduction

The surface electromyography (SEMG) is a valuable tool in the fields of biofeedback, prosthesis control, ergonomics, occupational and sports medicine, movement analysis, assessment of the neuromuscular functions, and diagnostic medicine [1,2,3]. Specifically, in occupational and sports medicine, it has been used widely to examine the occurrence of muscle contraction and relaxation, magnitude of muscle forces, as well as signal frequencies to quantify short-term and long-term muscle fatigue [4,5]. Indeed, the large volume of potent information that can be collected via SEMG measurement explains why many attempts have recently been made to use SEMG as a preferential functional technique that is embedded in a smart wearable system. Several products have already been developed and commercialized [6,7]. Representative examples include the Athos wearable system (Athos, USA) and Myontec’s Mbody technical shorts (Myontec Ltd, Kuopio, Finland). Both provide smart clothing in which SEMG electrodes are embedded with a visualized demonstration of real-time muscle activity. The validity of the SEMG values was accepted at a recreational level [8,9,10].

Despite those pioneers of smart clothing with SEMG electrodes on the market, there are still technological and socio-economical hurdles for the significant spread of smart clothing, such as high prices and aesthetical dissatisfaction, comfort, and ease of use [11,12,13]. At the same time, the rapid development of textile electrodes is producing sensor-embedded clothing that is like conventional clothing without deteriorating the wearer’s comfort [14,15]. In the current study, electrodes made of both carbon and silver layers were assessed in considering that they satisfy several requirements that Rodrigues et al. indicated in their study on titanium thin film-based electrodes: high corrosion, wear resistance, good mechanical ductility, chemical and thermal stability, and with high bio-compatibility [16]. In addition, a conductive sheet can be deposited over fabric with a heat press in a single step and electrodes and interconnector (electrical wire) can be produced at once. This may contribute to simplification and automation of the manufacturing process [17]. Moreover, heat pressed electrodes do not create unnecessary marks on the reverse side of the fabric. These characteristics correspond well with consumers’ requirements that technological electronics are not present externally in the clothing [11].

In designing SEMG electrode-embedded smart clothing, the electrode size is one of the important factors that should be determined carefully because it influences the SEMG signal quality [18,19]. In the case of traditional silver/silver chloride (Ag/AgCl) pregelled electrodes (Ag/AgCl electrodes), smaller electrodes are preferred theoretically, and the size of electrodes should not exceed 10 mm in the direction of the muscle fiber [20]. On the other hand, with regard to textile-based dry electrodes, most previous studies argue that larger s promise better signal quality along with decreased skin-electrode impedance [19,21,22]. An et al. [19] reported that the electrode-skin impedance of larger knitted electrodes (electrode’s surface area = 8 cm^2^) was less than one-third of that of smaller electrodes (electrode’s surface area = 2.25 cm^2^). Marozas et al. [21] demonstrated that textile-based electrodes with a surface area ≥4 cm did not cause significant distortion in the ECG signals in a low-frequency spectrum, whereas the 1 and 2 cm^2^ area electrodes distorted the signals at low frequencies. On the contrary, Puurtinen et al. [18] could not observe a gradual increase of electrode performance by increasing its size [18]. They described that the noise level of dry electrodes was practically the same in all sizes (Ø 7 mm to 30 mm) and only the largest one (Ø 30 mm) showed a lower noise level. In another aspect, larger electrodes are believed to be possibly less sensitive to slight differences in electrode positioning which can often occur during body movements [15]. Furthermore, larger electrodes hardly detect the specific muscle signals but nearby muscle groups, and may be easily affected by EMG crosstalk [23]. There have been concerns that covering the skin with a conductive film, usually low air- and water-permissible, may cause skin irritation problems, particularly with long-term use. Furthermore, any shift in the electrode location or wrinkles possibly caused by body movements can alter the contact area between the skin and electrodes, which may increase the number of movement artifacts. For this context, it would be necessary to identify the optimal size of the electrode.

Another valid issue is the impact of clothing pressure. In order for the sensor to be stably attached to the skin, it is necessary to make clothes of the right size. However, in order to design a “well-fitted” garment, not only the purpose of the garment (e.g., compression wear), comfort, but also the signal transmission performance at the electrodes must be considered [12]. In particular, consideration of signal transmission performance is necessary in order to provide accuracy to the smart clothing [12]. Clothing fit and pressure are important factors influencing clothing comfort, especially in tight-fit and compression sportswear [24]. Regional clothing pressure changes with interaction with the shape of the body, the fabric, and clothing design. Furthermore, the body posture can change clothing pressure [25]. In terms of the comfortable range of clothing pressure, Zhang et al. [26] performed a numerical simulation and suggested a range from 0 to 6 gf∙cm^–2^ (0 to 4.4 mmHg) as low pressure, and that between 15 and 25 gf∙cm^–2^ (11.0 and 18.4 mmHg) as a high-pressure zone. Kim and Lee [25] proposed a subjectively preferable range from 0.67 to 1.82 kPa (5.0 to 13.7 mmHg) because of human wearing trials of commercial compression sportswear. On the other hand, in the design of smart clothing functioning SEMG measurements, tighter-fit clothing is preferred because greater pressures imposed over the electrodes deform the skin and electrodes surface so that the contact area increases. An et al. [19] examined the pressure effect on SEMG recordings through textile electrodes. They reported an optimal clothing pressure of 30 mmHg because subjects started to feel uncomfortable at 30 mmHg. On the other hand, the current study aimed to find the optimal clothing pressure that shows a comparable performance to the traditional Ag/AgCl electrode within a lower range of clothing pressure. In the current study, clothing pressure was applied to the electrodes in a range from 6.1 to 12.6 mmHg, which was similar to the clothing pressure range measured for commercial compression sportswear by Kim and Lee [25].

The current study particularly aimed to investigate design factors that are particularly required in the designing and manufacturing process of smart clothing with SEMG electrodes. Among them, electrode size and clothing pressures over SEMG electrodes, which greatly influence SEMG signal were examined in vivo SEMG acquisition while strictly controlling each factor which can affect SEMG signal to clearly describe the effect of each factor. The analytic parameters for comparing the SEMG signal quality included the baseline EMG in the resting period indicating the electrode noise, average rectified EMG during muscle contraction, and signal-to-noise ratio (SNR) to compare the clearance of SEMG signal acquisition. Clothing pressure was adopted through the application of the pattern reduction rate (PRR) on the leg sleeves rather than adding thick foam between the electrodes and fabrics, considering that adjusting the PRR has less influence on the outward appearance of the clothing.

## 2. Materials and Methods

### 2.1. Electrode Preparation

**Figure 1:**
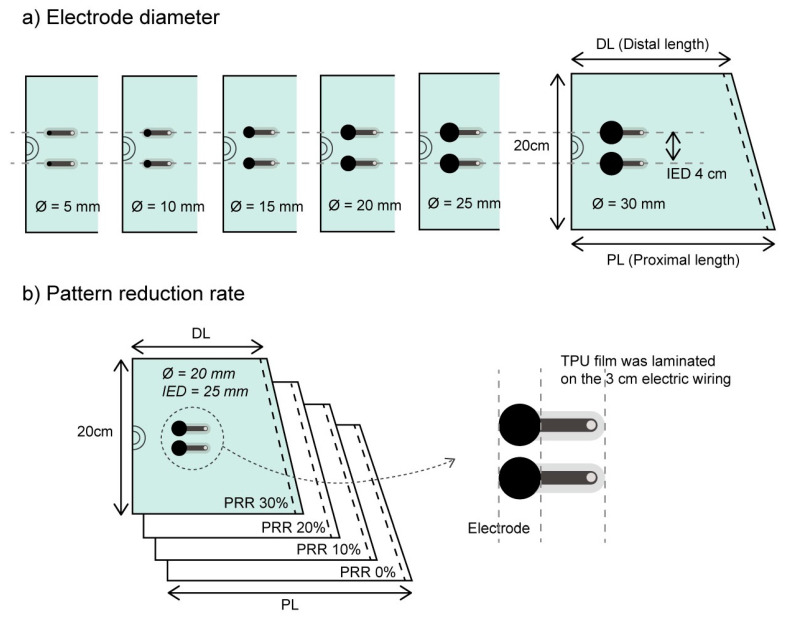
Electrode preparation in varied electrode diameters (**a**) and varied pattern reduction rate (PRR) (**b**). IED = inter-electrode diameter; PRR = pattern reduction rate. Three samples of each condition specification were prepared to reduce the sample error in the comparison.

In this study, textile-based electrodes were prepared in various sizes on leg sleeves (areal density; 245 g/m^2^, thickness; 0.402 mm, fiber content; polyester 76%, spandex 24%, Figure 1). The electrode was made from a conductive sheet composed of silver and carbon paste: The outer surface subject to direct contact with the skin was the carbon paste layer (surface resistance = 14.15 mΩ∙cm^2^). A silver paste layer was placed beneath it. All electrodes were trimmed in a circular form with an electric path (4 mm × 30 mm). The measurement leads of the SEMG device were connected to the textile-based electrodes using conductive snap fasteners, which were stuck at the end of each wiring. Three samples of each condition were prepared to determine the sample error. Except for the electrode area, other surfaces of conductive part exposed were all covered with a thermoplastic polyurethane (TPU) film to prevent EMG contamination by unintended signals.

The effects of the electrode size were examined by preparing six variations of electrode diameter, Ø 5–30 mm (Figure 1a). Two identical electrodes were attached along the fiber direction with a distance between their centers (IED, inter-electrode distance) equal to 40 mm. The IED was determined to avoid larger electrodes (Ø = 30 mm) overlapping or contacting each other.

**Figure 2:**
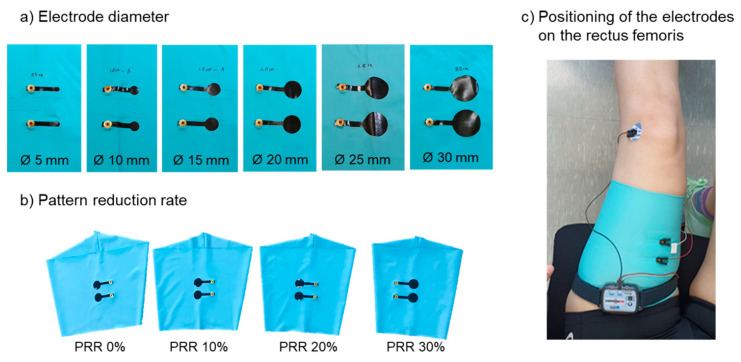
Actual SEMG electrodes in different diameters (**a**) and in different pattern reduction rates (**b**). Bipolar electrodes attached on a leg sleeve were positioned on the rectus femoris (**c**) and conductive snap fasteners were used to connect to the EMG acquisition system.

The effect of the clothing pressure was tested by preparing leg sleeves in different pattern reduction rates (PRR) from 0 to 30% (Figure 1b). The PRR was applied only in the width reduction of the leg sleeves and not in the length reduction. The distal length (DL) and proximal length (PL) were calculated using the following formula:

(1) $$\mathrm{Pattern}\;\mathrm{reduction}\;\mathrm{rate}\;\left(\%\right)=\;\frac{\left(DC-DL\right)}{DC}\times 100=\frac{\left(PC-PL\right)}{PC}\;\times 100$$

where *DC* is the distal circumference of the leg and *PC* is the proximal circumference of the leg. According to Formula (1), the leg sleeve of PRR 0% covered the leg with no elongation of the textile. The clothing pressure was measured for each PRR. The methods are described in Section 2.4. Regarding the samples in varied PRR, the electrode diameters were all 20 mm, and the inter-electrode distance was 25 mm. In all samples testing the effect of the clothing pressure, the other electric configurations were identical to the samples for testing the electrode sizes. Figure 2 shows the actual SEMG electrodes in different electrode diameters and pattern reduction rates which were used in the experiment.

### 2.2. EMG Measurement and Data Processing

The performance of the textile-based electrodes in detecting SEMG signals was validated by analyzing the EMG signal characteristics with two variables (electrode size and clothing pressure) during knee extension. A full-depth squat was also performed to test the clothing pressure effect. Bipolar EMG recordings were obtained using textile-based electrodes on the rectus femoris, which is located on the anterior thigh and is involved in knee extension and hip flexion. In this study, the rectus femoris was selected based on the following considerations. First, a relatively small muscle, such as biceps brachii, is not suitable for this test because it is recommended that the IED should not exceed 1/4 of the muscle-fiber length [27] (c.f. IED in this test was 25 and 40 mm). Second, the rectus femoris is located in the anterior thigh so that the electrodes can be placed stably during knee extension in the seated position, which minimizes the movement artifacts in the SEMG recordings. In this study, skin preparation before testing (e.g., shaving and exfoliating) was not performed, considering that people would not implement skin preparation when wearing SEMG suits in practical use [9]. The electrodes were positioned on the midpoint of the muscle belly for the rectus femoris. A reference electrode, pre-gelled self-adhesive Ag/AgCl electrodes (Kendall LTP, Covidien, MA, USA) was placed over the head of fibula, the electrically neutral bony prominence [28]. The recordings were amplified and filtered (20 to 500 Hz) in analog (MP160, BIOPAC Systems, Inc., Goleta, CA, USA). The data were full-wave rectified and averaged with a 100 ms time constant to draw the amplitude of the signals. Regarding the data obtained during a full-depth squat, they were also smoothed by 500 samples to extract a clear curved line. The entire data processing of SEMG was conducted using AcqKnowledge 5.0.1 Software (BIOPAC Systems, Inc., Goleta, CA, USA).

### 2.3. Experimental Protocol

**Figure 3:**
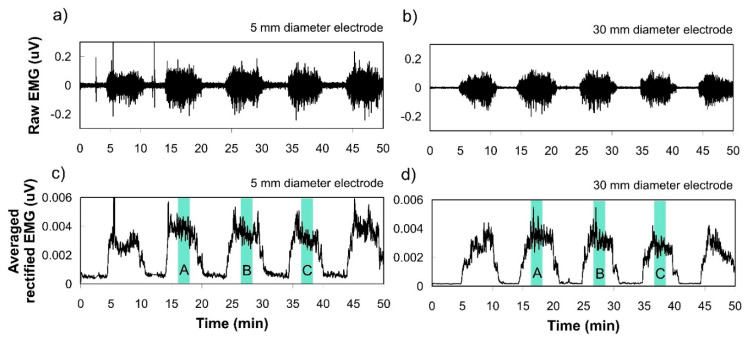
Examples of SEMG recordings from one subject during intermittent muscle contraction measured by 5 mm and 30 mm diameter electrodes. (**a**,**b**) Show an example of raw EMG signals obtained via electrode diameter Ø 5 mm and Ø 30 mm, respectively. (**b**) clearly shows lower baseline EMG than (**a**); (**c**,**d**) show filtered (20–500 Hz) in analog and full-wave rectified EMG to analyze the amplitude. Three contractions among five, except the first and last trials, were used to calculate the average activated EMG for comparison.

Before testing muscle contraction, the baseline EMG signals were collected in the supine position for 10 seconds to measure the baseline electrode noise. With the subject in the seated position, a single-joint knee extension was carried out to induce muscle contraction signals. The subject extended the knee until the lower leg became parallel to the floor. Testing consisted of five consecutive trials for knee extension and knee flexion (Figure 3). Each phase lasted for five seconds. To avoid the order effect, 18 trials (six conditions × three samples) for the size effect test and 12 trials (four conditions × three samples) for the pressure effect test were carried out in random order. After placing electrodes on the skin, the skin-electrode contact was allowed to stabilize for three minutes as the electrode-skin impedance decreased rapidly and stabilized in the first three minutes [19].

On the other hand, the current study was performed on a subject (sex = female, age = 32 years, height = 168 cm, body weight = 63 kg), so individual differences in the muscle activities were not considered. Instead, to distinguish the sample errors from the effects of testing conditions, all test conditions were done on three samples in an identical configuration (electrode sizes and clothing pressure). Calculation of the averages and standard deviations to identify the significance of the differences were based on three repeated measurements from three samples. Thus, sample errors could be determined [19]. During the entire experimental procedure, the room temperature was 25 °C, and the relative air humidity was 60%.

### 2.4. Clothing Pressure

An air-pack type contact pressure sensor was used to measure the clothing pressure imposed by leg sleeves with a different pattern reduction rate. The sensor (AMI 3037-2, AMI Techno, Co., Ltd., Tokyo, Japan) was placed on the rectus femoris, and covered directly by the EMG electrodes-embedded leg sleeve. The subject maintained standing postures for one minute to obtain stabilized values.

### 2.5. Data Analysis and Statistics

The baseline EMG signals were calculated by averaging five seconds of data. In comparison, EMG measurements during muscle contraction were calculated by averaging two seconds during five seconds of the activated phase. The signal to noise ratio (SNR) was calculated to present the signal power compared to the background electrode noise. Non-parametric statistics were used to verify the significance of between-group differences owing to insufficient sample size for parametric methods. The Kruskal–Wallis test was used first. When statistical significance was determined by the Kruskal–Wallis test, a Mann–Whitney U test was conducted as a post hoc test to distinguish statistically different pairs. Statistical significance was reported at a level of *p* < 0.1.

## 3. Results

### 3.1. Effect of the Electrode Diameter

**Figure 4:**
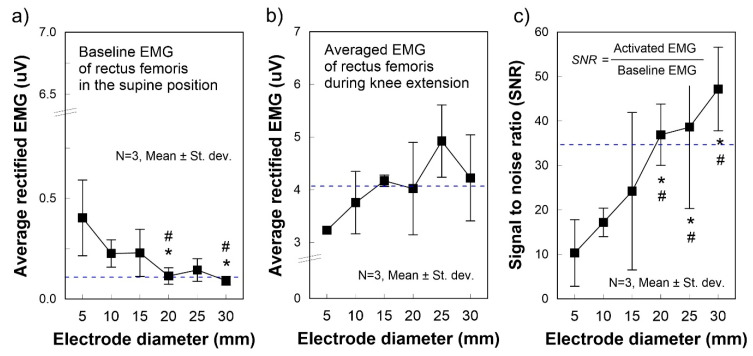
Average rectified SEMG of baseline and during muscle activation for different electrode diameters. The dashed lines indicate the values obtained from disposable Ag/AgCl electrodes. (**a**) Baseline electrode noise; (**b**) SEMG amplitude during muscle contractions exerted by knee extension; (**c**) Signal to noise ratio (SNR). SEMG was measured on the rectus femoris with stretchable leg sleeves where circle electrodes in different diameters were embedded. Values were calculated by averaging three consecutive measurements from three leg-sleeve samples in each diameter. * *p* < 0.1 versus 0%, # *p* < 0.1 versus 10%. Non-parametric statistics verified all significances: the Kruskal–Wallis test was used to identify group differences with the Mann–Whitney U test used as a post hoc test.

The baseline electrode noise decreased significantly with increasing electrode size (*p* = 0.073, Figure 4a). The Ø 20 and Ø 30 mm electrodes showed significant differences compared to those of Ø 5 mm and Ø 10 mm: (Ø 5 mm) 0.403 ± 0.188 μV; (Ø 10 mm) 0.266 ± 0.068 μV; (Ø 20 mm) 0.091 ± 0.022 μV; and (Ø 30 mm) 0.114 ± 0.041 μV (*p* < 0.1). The baseline noise of the pre-gelled self-adhesive Ag/AgCl electrode was 0.118 μV, so the Ø 20 mm and Ø 30 mm electrodes showed comparable baseline electrode noise levels to the Ag/AgCl electrodes. Regarding the EMG signals during muscle contraction (Figure 4b), an increasing tendency for larger electrodes was observed, but the between-group difference was not statistically significant. The EMG during muscle contraction from the Ø ≥ 15 mm electrodes was similar to or marginally larger than the Ag/AgCl electrodes. On the other hand, the SNR consistently and significantly increased with increasing electrode size (*p* = 0.053, Figure 4c). Electrodes Ø ≥ 20 mm showed significant differences compared to the Ø 5 and Ø 10 mm electrodes (*p* < 0.1).

### 3.2. Effect of the Clothing Pressure

The clothing pressure increased gradually with increasing PRR: 6.1, 8.1, 10.1, and 12.6 mmHg for the PRR, and 0, 10, 20, and 30%, respectively. The range of clothing pressure corresponded approximately to the range that Kim and Lee [25] reported in the investigation on the clothing pressure of the commercial compression sportswear, 5.0–13.7 mmHg.

**Figure 5:**
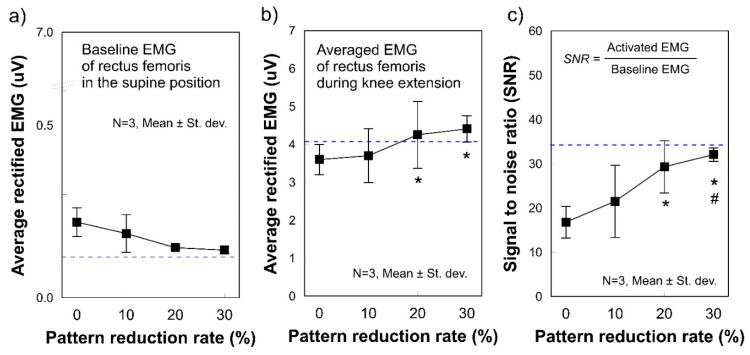
Average rectified SEMG of the baseline and during muscle activation for different pattern reduction rates. (**a**) baseline electrode noise and (**b**) SEMG amplitude during muscle contractions exerted by knee extension; (**c**) Signal to noise ratio (SNR). SEMG was measured on the rectus femoris with stretchable leg sleeves where circle electrodes with different diameters were embedded. The values were calculated by averaging three consecutive measurements from three leg-sleeve samples in each diameter. * *p* < 0.1 versus 0%, # *p* < 0.1 versus 10%. Non-parametric statistics verified all significances. The Kruskal–Wallis test was used to identify group differences with the Mann-Whitney U test used as a post hoc test.

During knee extensions, SNR consistently and significantly increased with increasing PRR (*p* = 0.066, Figure 5c). Electrodes with PRR 20% and 30% showed significant differences compared to PRR 0%, whereas PRR 30% showed a significant difference compared to PRR 10% (*p* < 0.1). A decreasing tendency was observed in the baseline EMG signals (Figure 5a), and an increasing tendency was noted in the activated EMG signals (Figure 5b), but there the differences were not significant. Nevertheless, the electrodes of PRR ≥ 20% are preferred because the activated EMG for the electrodes of PRR ≥ 20% (*P_c_* = 10.1 mmHg) was marginally greater than that of the Ag/AgCl electrodes (Figure 5b). In addition, the SNRs for the electrodes of PRR ≥ 20% were significantly greater than those for the electrodes of PRR 0 and 10% (*p* < 0.1). When preparing clothing pressure substitutes for the PRR, a clothing pressure ≥ 10.1 mmHg can be recommended considering its comparable performance to the Ag/AgCl electrodes.

**Figure 6:**
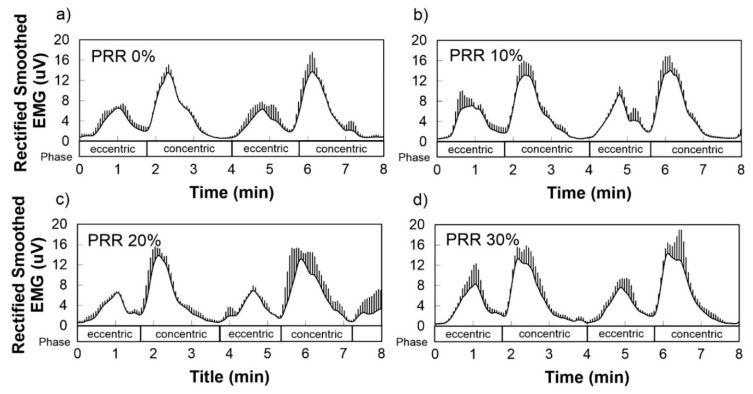
Average rectified smoothed SEMG during a full-depth squat. The electrodes were embedded on the leg sleeves with a varied pattern reduction rate (PRR) of (**a**) 0%, (**b**) 10%, (**c**) 20%, and (**d**) 30% so that the muscle activities of the rectus femoris were recorded. The values were averaged from three leg-sleeve samples in each PRR.

The EMG measurement during full-depth squat showed no significant differences by PRR or clothing pressure. Figure 6 represents muscle contractions of the rectus femoris during eccentric and concentric phases, but during all phases, significant differences in their amplitudes were not detected among the four conditions (*p* > 0.1).

## 4. Discussion

The current study examined the optimal electrode size and clothing pressures over SEMG electrodes through in vivo SEMG acquisition. The results showed that the baseline noise decreases with increasing electrode diameter and the signal to noise ratio (SNR) increased with decreasing electrode diameter and increasing clothing pressure along with the comparative performance of textile-based electrodes: Ø ≥ 20 mm and *P_c_* ≥ 10 mmHg compared to the Ag/AgCl electrodes. In addition, the SEMG signals during full-depth squat indicated that the effect of clothing pressure would be reduced during exercises causing the body circumferences to change. The results offer a meaningful suggestion for the optimal design of EMG suits with electrodes for the current electrode material. Moreover, the methodology suggests a part of the development process to accomplish smart wear with high accuracy.

In the process of electrode design optimization, the electrode size was considered one of the most significant factors influencing the SEMG signal quality. In previous studies [19,21,22], larger dry electrodes promised lower electrode-skin impedance and better EMG signal quality which was evaluated by electrode-skin impedance [19,22] and spectral analysis [21]. On the other hand, Puurtinen et al. [18] showed results which were not completely consistent regarding dry electrodes with above previous studies [19,21,22] and described that the noise level of dry electrodes was practically the same in all sizes (Ø 7–30 mm) and only the largest one (Ø 30 mm) showed a lower noise level. The current results support the lowered baseline noise level and greater SNR with larger electrodes as most previous studies [19,21,22], which is possibly attributed to controlling the pressure over the electrode because the interface between skin and dry electrodes is less stable than gelled or wet electrodes [18,19].

Despite such results revealing a relationship between the electrode size and signal quality, it is difficult to say that the electrode size and clothing can be designed based simply on those outcomes as the SEMG signal acquisition can differ according to the electrode material because the electric conductivity and the surface morphology vary. To achieve smart clothing with high accuracy, the performance should be evaluated in each electrode configuration. The textile-based electrodes used in this study showed acceptable performances in SEMG signal acquisition, even when compared to Ag/AgCl electrodes, when the electrode diameter and clothing pressure were Ø ≥ 20 mm and *P_c_* ≥ 10 mmHg, respectively.

In this study, clothing pressure was applied over the electrodes in the range of 6.1–12.6 mmHg. This is the range that wearers can feel comfort and clothing contact with the skin. Generally, two approaches can possibly be able to impose clothing pressure over the electrode. The first involves adjusting the clothing tightness using arm- or leg-sleeves [19]. Second, pads or foams of various thicknesses can be inserted between the electrodes and the substrate fabrics [29,30]. The latter method can be applied in less tight fitting clothing. On the other hand, it can alter the clothing silhouette by making uneven surfaces of clothing. The current study adopted varied clothing pressures by adjusting the size of the leg sleeves with various PRR, which was also done by An et al. [19]. This method produces less distortion of the clothing silhouette.

The most important question here can be, “What is the optimal clothing pressure for EMG suits?” An et al. [19] compared EMG signals with varying clothing pressures from 5 to 45 mmHg and proposed 30 mmHg because the subjects started to feel uncomfortable at 30 mmHg (4 kPa). Cömert et al. [30] also tested the pressure effect on ECG recordings by applying electrode pressures between 5 and 25 mmHg. They suggested 15–20 mmHg as the appropriate pressure to reduce motion artifacts. In this study, clothing pressure over an electrode of more than 10 mmHg for a textile-based electrode (Ø 20 mm) was classified as the acceptable range because the EMG signal during muscle contraction was comparable to a conventional Ag/AgCl electrode. The motion artifacts were not considered in this study. The emphasis was placed on the electrode noise and EMG amplitude during muscle contraction. Nevertheless, from the data during the full-depth squat, a clear curved line was observed without significant motion artifacts.

This study explored a method to improve SEMG signal quality by modulating clothing pressure and electrode size, and a comparable performance to Ag/AgCl electrodes was attained by increasing electrode diameter and clothing pressure. In particular, the current study suggests that obtaining better performance with a smaller electrode can be achieved by increasing PRR, thus clothing pressure. For instance, electrode Ø 10 mm can show better performance than the results (Figure 4) with a greater PRR than 20% or more because PRR 20% was used in the electrode diameter change experiment. To further improve the signal quality, other approaches with respect to materials and surface morphology to reduce the skin-electrode impedance may work [14]. Furthermore, advanced filtering processes can help to make the SEMG signals much clearer by removing unintended signals.

However, it should be noted that when reducing the electrode size, it is necessary to additionally confirm whether little or no displacement of the electrode occurs over the clothing. However, displacement of electrodes and the skin may often occur due to body movement or wrinkles of the textile, and it greatly interferes with the EMG signal collection. Finni et al. [15] used larger electrodes to measure EMG through clothing with consideration that the larger conductive area is not so sensitive to slight differences in electrode positioning. It is almost impossible to use a method that completely adheres to the skin such as Ag/AgCl in smart clothing. Hence, when reducing the electrode size, more effort is required to stably attach the electrode to the skin.

With regard to the methodology, skin adhesion is a required process to obtain satisfactory electrode-skin impedance. This normally includes shaving, removing the skin stratum corneum, and cleaning with alcohols or skin prep gels. Lu et al. [31] reported the significance of skin abrasion even in a dry electrode due to the instability of the skin-electrode contact area. This was supported by a change in skin-electrode impedance with wet electrodes. In this study, however, no measure for skin adhesion has been adopted before testing because it is hardly expected that the person who wears textile-based electrodes-embedded clothing will perform the steps for skin preparation to improve the quality of the SEMG signals. A similar level of skin preparation would be meaningful to verify the accuracy and validity of the SEMG acquisition during daily use, even though it could increase the electrode-skin impedance.

The manufacturing process of smart clothing, especially the integration process between textile components and electronic components tends to be complex (Dunne et al., 2010). The automation of manufacturing has been advancing, which is expected to accelerate the commercialization and popularization of smart clothing by reducing manufacturing costs and thus product price. As a conductive sheet can be deposited over fabric with a heat press in a single step, the manufacturing process can be simplified and most processes can be automated [13]. In addition, the electrode and interconnection, electrical wires, can be designed in a connected form, which may allow simplification of the production process and increase durability by reducing the number of connections between components [7]. However, further studies are needed for actualization of automating the manufacturing process for smart clothing. Studies should encompass various aspects including clothing pattern design for fine ready-to-wear products and on-demand produced clothing.

## 5. Conclusions

The current results suggest the design optimization for textile-based SEMG electrodes and clothing in which they are embedded. The effects of electrode size and clothing pressure over electrodes on SEMG signals were analyzed based on in vivo SEMG acquisition during leg extension and full-depth squat. The following can be suggested as a conclusion of this study:

(1) Greater electrode contact area presented better EMG signals by showing a decreased baseline noise and a greater SNR. However, finding the optimal electrode size while balancing between the performance of SEMG acquisition (larger would be better) and SEMG crosstalk and cost (smaller would be better) is recommended.

(2) Clothing pressure over an electrode of more than 10 mmHg for a textile-based electrode (Ø 20 mm) was classified as an acceptable range. During a full-depth squat, the effect of clothing pressure was even diminished. However, an optimal pressure can be altered with different surface characteristics of the electrode, especially linked to the stability of skin-electrode contact.

(3) Textile-based electrodes used in the current study denoted comparable performance to Ag/AgCl electrodes when Ø ≥ 20 mm and Pc ≥ 10 mmHg. These results may be due to the similar textile-based electrodes made from a conductive sheet composed of silver and carbon layers like the current study, but not for the other electrodes fabricated by a disparate method because the optimal design of electrodes would differ according to the electrode material and the purpose of use.

(4) The current results emphasize that comparable performance with Ag/AgCl electrodes can be obtained by modulating electrode size and pressures even with a textile-based electrode. Along with the improvement of electrode materials and contact morphology to reduce skin-electrode impedance, further studies on the sophisticated clothing pattern making to secure reasonable clothing pressure while minimizing an electrode displacement would contribute to developing a better clothing-type SEMG acquisition system.

## Abbreviations

DC	Distal circumference
DL	Distal length
ECG	Electrocardiography
EMG	Electromyography
EOG	Electrooculography
IED	Inter-electrode distance
PC	Proximal circumference
*P_c_*	Clothing pressure
PL	Proximal length
PSD	Power spectral density
PRR	Pattern reduction rate
RMS	Root mean square
SEMG	Surface electromyography
SNR	Signal to noise ratio
TPU	Thermoplastic polyurethane

## References

[1] Cram J.R., Steger J.C. (1983). EMG Scanning in the diagnosis of chronic pain. Biofeedback Self Regul..

[2] Kleissen R.F.M., Buurke J.H., Harlaar J., Zilvold G. (1998). Electromyography in the biomechanical analysis of human movement and its clinical application. Gait Posture.

[3] Subbu R., Weiler R., Whyte G. (2015). The practical use of surface electromyography during running: Does the evidence support the hype? A narrative review. BMJ Open Sport Exerc. Med..

[4] Luttmann A., Jäger M., Laurig W. (2000). Electromyographical indication of muscular fatigue in occupational field studies. Int. J. Ind. Ergon..

[5] Siyeon K., Jeong W. (2020). Physiological and psychological neck load imposed by ballistic helmets during simulated military activities. Fash. Text..

[6] Giminiani R.D., Cardinale M., Ferrari M., Quaresima V. (2020). Validation of fabric-based thigh-wearable EMG sensors and oximetry for monitoring quadriceps activity during strength and endurance exercises. Sensors.

[7] Sayem A.S.M., Teay S.H., Shahariar H., Fink P.L., Albarbar A. (2020). Review on smart electro-clothing systems (SeCSs). Sensors.

[8] Bengs D., Jeglinsky I., Surakka J., Hellsten T., Ring J., Kettunen J. (2017). Reliability of measuring lower-limb-muscle electromyography activity ratio in activities of daily living with electrodes embedded in the clothing. J. Sport Rehabil..

[9] Lynn S.K., Watkins C.M., Wong M.A., Balfany K., Feeney D.F. (2018). Validity and reliability of surface electromyography measurements from a wearable athlete performance system. J. Sports Sci. Med..

[10] Aquino J., Roper J. (2018). Intraindividual variability and validity in smart apparel muscle activity measurements during exercise in men. Int. J. Exerc. Sci..

[11] Ju N., Lee K.-H. (2020). Consumer resistance to innovation: Smart clothing. Fash. Text..

[12] Fernándex-Caramés T.M., Fraga-Lamas P. (2018). Towards the internet-of-smart-clothing: A review on IoT wearables and garments for creating intelligent connected e-textiles. Electronics.

[13] Dunne L. (2010). Smart clothing in practice: Key design barriers to commercialization. Fash. Pract..

[14] Acar G., Ozturk O., Golparvar A.J., Elboshra T.A., Bohringer K., Yapici M.K. (2020). Wearable and flexible textile electrodes for biopotential signal monitoring: A review. Electronics.

[15] Finni T., Hu M., Kettunen P., Vilavno T., Cheng S. (2020). Measurement of EMG activity with textile electrodes embedded into clothing. Physiol. Meas..

[16] Rodrigues M.S., Fiedler P., Küchler N., Domingues R.P., Lopes C., Borges J., Haueisen J., Vaz F. (2020). Dry electrodes for surface electromyography based on architecture titanium thin films. Materials.

[17] Dabby N., Aleksov A., Lewallen E., Oster S., Fygenson R., Lathrop B., Bynum M., Samady M., Klein S., Girouard S. A scalable process for manufacturing integrated, washable smart garments applied to heart rate monitoring. Proceedings of the 2017 ACM International Symposium on Wearable Computers (ISWC ’17).

[18] Puurtinen M.M., Komulainen S.M., Kauppinen P.K., Malmivuo J.A.V. Measurement of noise and impedance of dry and wet textile electrodes, and textile electrodes with hydrogel. Proceedings of the 2006 International Conference of the IEEE Engineering in Medicine and Biology Society.

[19] An X., Tangsirinaruenart O., Stylios G.K. (2019). Investigating the performance of dry textile electrodes for wearable end-uses. J. Text. Inst..

[20] Merletti R., Hermens H.J., Merletti R., Parker P. (2004). Detection and conditioning of the surface EMG signal. Electromyography—Physiology, Engineering, and Noninvasive Applications.

[21] Marozas V., Petrenas A., Daukantas S., Lukosevicius A. (2011). A comparison of conductive textile-based and silver/silver chloride gel electrodes in exercise electrocardiogram recordings. J. Electromyogr..

[22] Li G., Wang S., Duan Y.Y. (2017). Towards gel-free electrodes: A systematic study of electrode-skin impedance. Sens. Actuator B Chem..

[23] Mesin L. (2020). Crosstalk in surface electromyogram: Literature review and some insights. Phys. Eng. Sci. Med..

[24] Wong A.S.W., Li Y., Zhang X. (2004). Influence of fabric mechanical property on clothing dynamic pressure distribution and pressure comfort on tight-fit sportswear. Sen’i Gakkaishi.

[25] Kim N.Y., Lee H. (2019). Influence of clothing pressure on blood flow and subjective sensibility of commercial sports compression wear. Fash. Text. Res. J..

[26] Zhang X., Yeung K.W., Li Y. (2002). Numerical simulation of 3D dynamic garment pressure. Text. Res. J..

[27] Standards for Surface Electromyography: The European Project “Surface EMG for Non-Invasive Assessment of Muscles (SENIAM)”. http://citeseerx.ist.psu.edu/viewdoc/download?doi=10.1.1.623.2040&rep=rep1&type=pdf.

[28] De Silva J.C.L., Tarassova O., Ekbolom M.M., Andersson E., Rönquist G., Arndt A. (2016). Quadriceps and hamstring muscle activity during cycling as measured with intramuscular electromyography. Eur. J. Appl. Physiol..

[29] Pani D., Achilli A., Spanu A., Bonfiglio A., Gazzoni M., Botter A. (2019). Validation of polymer-based screen-printed textile electrodes for surface EMG detection. IEEE Trans. Neural Syst. Rehabil. Eng..

[30] Cömert A., Honkala M., Hyttinen J. (2013). Effect of pressure and padding on motion artifact of textile electrodes. Biomed. Eng. Online.

[31] Lu F., Wang C., Zhao R., Du L., Fang Z., Guo X., Zhao Z. (2018). Review of stratum corneum impedance measurement in non-invasive penetration application. Biosensors.

